# A Smartphone App to Assess Alcohol Consumption Behavior: Development, Compliance, and Reactivity

**DOI:** 10.2196/11157

**Published:** 2019-03-25

**Authors:** Antoinette Poulton, Jason Pan, Loren Richard Bruns Jr, Richard O Sinnott, Robert Hester

**Affiliations:** 1 Melbourne School of Psychological Sciences University of Melbourne Parkville Australia; 2 Melbourne eResearch Group School of Computing and Information Services University of Melbourne Parkville Australia

**Keywords:** alcohol drinking, smartphone apps, smartphone, mobile phone, research app development, compliance, reactivity

## Abstract

**Background:**

There are disadvantages—largely related to cost, participant burden, and missing data—associated with traditional electronic methods of assessing drinking behavior in real time. This potentially diminishes some of the advantages—namely, enhanced sample size and diversity—typically attributed to these methods. Download of smartphone apps to participants’ own phones might preserve these advantages. However, to date, few researchers have detailed the process involved in developing custom-built apps for use in the experimental arena or explored methodological concerns regarding compliance and reactivity.

**Objective:**

The aim of this study was to describe the process used to guide the development of a custom-built smartphone app designed to capture alcohol intake behavior in the healthy population. Methodological issues related to compliance with and reactivity to app study protocols were examined. Specifically, we sought to investigate whether hazard and nonhazard drinkers would be equally compliant. We also explored whether reactivity in the form of a decrease in drinking or reduced responding (“yes”) to drinking behavior would emerge as a function of hazard or nonhazard group status.

**Methods:**

An iterative development process that included elements typical of agile software design guided the creation of the CNLab-A app. Healthy individuals used the app to record alcohol consumption behavior each day for 21 days. Submissions were either event- or notification-contingent. We considered the size and diversity of the sample, and assessed the data for evidence of app protocol compliance and reactivity as a function of hazard and nonhazard drinker status.

**Results:**

CNLab-A yielded a large and diverse sample (N=671, mean age 23.12). On average, participants submitted data on 20.27 (SD 1.88) out of 21 days (96.5%, 20.27/21). Both hazard and nonhazard drinkers were highly compliant with app protocols. There were no differences between groups in terms of number of days of app use (*P*=.49) or average number of app responses (*P*=.54). Linear growth analyses revealed hazardous drinkers decreased their alcohol intake by 0.80 standard drinks over the 21-day experimental period. There was no change to the drinking of nonhazard individuals. Both hazard and nonhazard drinkers showed a slight decrease in responding (“yes”) to drinking behavior over the same period.

**Conclusions:**

Smartphone apps participants download to their own phones are effective and methodologically sound means of obtaining alcohol consumption information for research purposes. Although further investigation is required, such apps might, in future, allow for a more thorough examination of the antecedents and consequences of drinking behavior.

## Introduction

### Background

Advantages associated with real-time (or near real-time) methods of assessing alcohol consumption behavior for research purposes have been widely documented in recent years [1-3]. Such methods—which are increasingly electronic in nature—are advocated on the basis of how they allow data to be captured repeatedly in the natural environment and in the absence of the researcher [2,3]. This facilitates the collection of actual intake information rather than the summary data commonly elicited from more traditional retrospective methods of assessing drinking. As such, recall and response biases are thought to be minimized and the ecological validity of the data is consequently enhanced [3]. Real-time methods additionally reduce the quantity of missing data and can yield a diverse and potentially very large sample [4,5]. Crucially, real-time data enable variations in behavior to be examined across time and in concert with cognitive, affective, environmental, and physiological factors. In this way, the antecedents and consequences of drinking behavior can also be investigated [2,3].

Real-time electronic methods of collecting alcohol consumption information for research purposes have evolved rapidly over the last two decades. Early studies typically featured hand-held electronic devices [6,7] or interactive voice response systems [8,9]. In the case of the former, participants used the device as an electronic diary; in the latter, the interactive voice response system was programmed to call participants on researcher-supplied cellular phones so that an automated questionnaire could be administered. Short message service (SMS) text messaging protocols have also been employed. In such studies, SMS text messages direct participants to follow a link to complete a Web-based survey via their mobile browser [10] or ask them to respond to simple questions about their alcohol consumption via text [11]. With the advent of smartphones, researchers have increasingly been programming study-specific apps for use in such studies. In most cases, this involves either providing participants with a phone preloaded with the app [12,13] or loading the app onto participants’ own phones and downloading the data at the end of the experimental period [14].

There are a number of disadvantages associated with utilizing the aforementioned electronic protocols. There are costs, for instance, associated with programming, supplying, and training participants to use electronic devices that are not their own [15-18]. Participants must also be provided with some means whereby they can recharge these devices [16]. If they have their own mobile, they will be carrying 2 devices during the experimental period, which might prove unduly burdensome and reduce compliance. Questionnaires completed via cell phone browsers do not always scale well to all mobile devices and require internet connectivity [19]. Surveys conducted via SMS text messages are necessarily limited in scope and, in the absence of internet connectivity, potentially costly to participants [20]. In all cases, participants are required to visit the lab at least twice during the experimental period; they must collect the device or have the program loaded onto their own phone plus undergo training, and they must return the device or have the data downloaded off their phone [18,21]. These disadvantages potentially diminish some of the benefits of using electronic devices to collect real-time alcohol intake behavior; specifically, sample size and diversity may be a function of the cost of supplying the device to those willing and able to attend the lab, and data may go unrecorded where internet connectivity or cost to the participant is an issue.

Using apps participants download to their own smartphones, without ever visiting a lab, might enable researchers to more fully realize the benefits of collecting alcohol consumption information electronically and in real time. Smartphone penetration currently stands at upward of 70% across many developed nations and is growing rapidly across developing ones [22]. Across the United States, United Kingdom, and Australia, more than 93% of 18 to 34 year olds own a smartphone [23]. Taking advantage of high smartphone ownership rates by using participants’ own devices represents a substantial cost reduction both in terms of equipment and training. Asking individuals to download apps via marketplace vendors and having data stored on the phone until it can be automatically uploaded to a server via a Wi-Fi connection alleviates the participants’ need to visit the lab and reduces the likelihood of data being lost or going unrecorded. Advantages of real-time assessment related to completeness of data, sample size, and diversity might therefore be preserved in studies employing apps downloaded to participants’ smartphones.

Nonetheless, there are a number of development and methodological concerns pertaining to assessing alcohol consumption behavior via smartphone apps that warrant further investigation. Given that any app must be programmed for 2 different, frequently updating operating systems with distinct deployment protocols, researchers are likely to require the assistance of 1 or more app programmers in the development phase. The literature offers little guidance regarding the software development process as it relates to the behavioral sciences [24]. Examples of how to effectively manage and integrate the requirements and expectations of multiple stakeholders when developing apps for behavioral studies are consequently required.

Although several recent studies have reported promising results with regard to the validity of app-based methods of capturing alcohol intake information [25-27], issues related to app protocol compliance and reactivity have received less attention. Compliance refers to the extent to which participants adhere to study requirements and protocols throughout the experimental period, whereas reactivity describes a process whereby the monitoring of a behavior results in a change in that behavior [1-3]. Simply completing traditional alcohol intake assessment and screening measures has been found to decrease consumption among heavy drinkers and reduce self-reported hazardous drinking behaviors [28-30]. However, it is unclear if this reactivity arises as a function of drinking behavior. Individuals characterized by more risky consumption patterns may, more likely than others, alter their behavior in response to monitoring. Although commentators generally report little evidence of reactivity in real-time studies of alcohol intake, they also note further investigation is required [1,21,31-33]. Reactivity is a phenomenon that can emerge for several reasons. Participants might become more aware of the behavior and become consequently motivated to implement change, or the demands of the protocol might provoke a tendency to satisfice [31]. In any real-time study of alcohol intake, a decrease in drinking could be evidence of the former, whereas a decrease in responding “yes” to the behavior (and therefore in having to respond to further questions) might suggest the latter.

### Objectives

In this paper, we describe the process used to guide the development of a custom-built smartphone app designed to capture alcohol intake behavior in the healthy population for research purposes. We also evaluate methodological issues related to compliance with and reactivity to study protocols as a function of hazard and nonhazard drinker status. We expect individuals in both hazard and nonhazard groups will be equally compliant. Reactivity in the form of a decrease in drinking will be minimal or confined to the hazard group, whereas individuals in both groups will be susceptible to reactivity in the form of reduced responding.

## Methods

### App Development

The CNLab-A app represents the outcome of an iterative development process that included elements of agile software design, namely requirements analysis, feature and interface design, and app implementation [24,34].

#### Requirements Analysis

In the requirements analysis phase, the research team determined key variables of interest. To this end, empirical definitions of excessive and binge drinking and variables derived from validated retrospective measures of drinking were examined [35-39]. We aimed to elicit data that would assist in establishing percentage of drinking and nondrinking days, daily and average total standard drinks, daily and average drinking rate, highest drink count in 2 hours, and blood alcohol content (BAC). After soliciting advice from the programmer and giving due consideration to the ethical implications, we decided that although the date and time of app submissions would be automatically logged, geolocation would not be recorded because of concerns that such data may undermine efforts to preserve user anonymity [40,41]. In early iterations of the app, responses related to date of birth, sex, height, and weight were also recorded. Date of birth and sex details were utilized to link data from the app with information collected via other means (eg, Web-based surveys or in the lab). Height and weight were used to determine BAC. In this early iteration, all data were uploaded to a dedicated commercial Web-server company account. Given the concerns articulated in the literature regarding privacy and the secure storage of behavioral data [40,41], latter iterations of the app did not include any demographic questions; instead, a unique identification number was generated for each participant, and the data were uploaded to a secure server. In this way, app data were not linked to any personal information.

In this phase, the research team also considered assessment design. Studies reliant on real-time (or near real-time) monitoring of behavior tend to employ event- or time-based sampling [32]. In the case of the former, participants record the behavior as it happens or shortly thereafter; in the latter, behavior is recorded in response to signals that occur multiple times a day, often at random [32]. Occasionally, both assessment methods are utilized simultaneously [32]. As substance use is episodic in nature, event-based assessment is considered an appropriate method for tracking both frequency and timing of use [1]. Therefore, we determined this would be the most pertinent method of collecting alcohol consumption information. However, a disadvantage of event-based monitoring is that it is difficult to assess compliance, that is, there is no way to verify that participants record all events as required [32]. To combat this limitation, we required the app to send participants twice-daily prompts asking them if they had consumed alcohol since the last submission. This served to remind participants to record drinking if they had forgotten to do so. In this regard, we adopted a similar assessment protocol as Dulin and colleagues; they also employed an event-based assessment along with daily notifications reminding participants to record drinking [25]. We additionally required the app to prevent participants from submitting data more than 24 h into the past or more than 15 min into the future to prevent back and forward filling.

**Figure 1:**
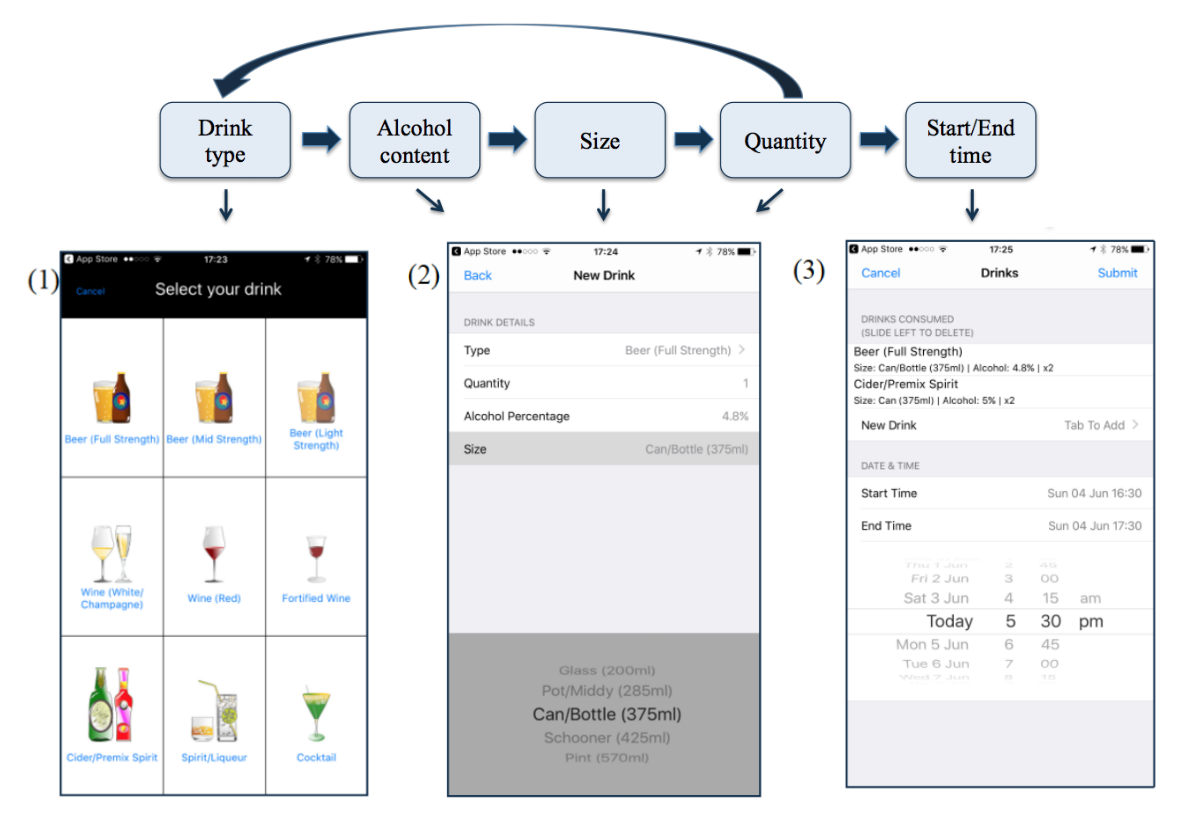
Flowchart of assessment pathway and screenshots from the CNLab-A app. On opening, CNLab-A asks users if alcohol has been consumed in the last 24 hours. Thereafter, participants are asked if they have consumed alcohol since their last submission. If they indicate (by pressing “No”) that no drinking has occurred, the app can be closed. If participants indicate drinking has occurred (by pressing “Yes”), images of common alcoholic beverages (including beer, wine, cider/premix, spirit/liqueur, and cocktail) are displayed (1). Type of beverage consumed is selected by touching the appropriate image on the screen. Quantity and size consumed for each beverage is indicated via a simple scroll option menu (2). Alcohol content as a function of beverage type is prefilled. This process is repeated by tapping “Back” in order to add as many drink types as required. Erroneously entered data can be deleted by swiping left. Prior to submitting data, the start and end time of drinking must be specified, again using a scroll option menu (3). Participants are able to either report drinking in separate sessions or they can leave the app open so as to record beverages as they are consumed. The latter option still allows participants to use other features on their phone. Participants can access a history of their submission dates and times (but not their drinking data) via the “History” button. At the conclusion of the experimental period, an automated message thanks participants; gives them simple feedback regarding the number of days they consumed alcohol, total standard drinks consumed, and average daily consumption; and, asks them to remove the app from their smartphone.

Finally, the content for each assessment was based on the key variables of interest. Drink type formed the central element of each assessment as, once selected, all subsequent items were dependent on this choice (Figure 1). For instance, selecting mid strength beer as the drink type in the app automatically determined the alcohol content and serving size options. In Australia, mid strength beer has an alcohol content of 3.5% and is available, at least in licensed and retail venues, in various standard serving sizes [42,43]. Table 1 details drink type, alcohol content, and serving size options available in the app (Table 1). These options were not meant to be exhaustive but were designed to capture typical drink types, average alcohol content, and standard serving sizes sold in Australia. The final aspect of assessment involved selecting a start and finish time for drinking.

Decisions made during the requirements analysis phase were documented via an iterative storyboard process. This ensured that the research team and programmer had access to a common record.

#### Feature and Interface Design

Feature and interface design was informed by app software capabilities and in consultation with the programmer. During this phase, iOS app prototypes were developed and deployed in response to design decisions and feedback from the research team. Deployment was via Test Flight, which allowed invited users to test beta versions of iOS apps.

We determined response selection would be via touch screen and scroll menus. At the commencement of each assessment, for instance, when participants were asked if they have consumed alcohol since their last submission, they indicated “Yes” or “No” by touching either option on the screen. Similarly, it was decided that drink types would be presented on 1 touch screen as a set of images with captions so that participants could readily identify and select their beverage. Images were designed using Inkscape, a freely available vector graphic software package. In the case of beer, images for mid and light-strength were opaque versions of the full-strength visual. Quantity and size options were available via a simple scroll option menu. Likewise, a scroll menu presented dates and times (in 15-min intervals) for recording of drinking start and end time. Participants pressed “Submit” to upload (to the server) and end the assessment. Any submission that failed to upload because of lack of internet connectivity would automatically be uploaded when connectivity was reestablished. Figure 1 shows screenshots of the app interface (Figure 1). The app icon was also designed during this phase. A discreet pattern and app name were chosen to minimize the likelihood friends or family of the participant would realize they were taking part in an alcohol-related study.

#### App Implementation

One of the potential advantages of using electronic means of assessment in the behavioral sciences relates to penetration. Such methods can provide investigators with large, diverse samples if facilitated by appropriate implementation decisions related to development and deployment. To that end, the research team decided to develop a native app to be run locally on iOS and Android platforms with marketplace deployment via Apple iTunes and Google Play, respectively, to optimize app availability. However, because of funding limitations, only an iOS version of the app was initially alpha and beta tested.

**Table 1:** Drink types, alcohol content, and serving size options available in the CNLab-A app.

Drink type		Alcohol content (%)	Serving sizes
**Beer**			
	Full strength	4.8	Glass 200 mL; pot/middy 285 mL; can/bottle 375 mL; schooner 425 mL; pint 570 mL
	Mid strength	3.5	As above
	Light strength	2.7	As above
**Wine**			
	White/Champagne	12.0	Glass 150 mL
	Red	13.0	As above
**Fortified wine**			
	Port, sherry, marsala, madeira, vermouth, etc	18.0	Glass 60 mL
**Cider/premix spirit**			
	Most ciders and premix drinks including alcopops	5.0	Bottle 300 mL; bottle 330 mL; can 375 mL; bottle 500 mL
**Spirit/liqueur**			
	Rum, gin, vodka, brandy, tequila, whiskey, liqueurs, etc	40.0	Standard 30 mL; double 60 mL
**Cocktail**			
	Various	40.0	1 shot 30 mL; 2 shots 60 mL; 3 shots 90 mL; 4 shots 120 mL

Alpha testing involved a small sample of individuals (N=8, mean age 38.13 years, SD 16.54; range 18-68 years, 37.5% female) and was designed to identify programming bugs and oversights. Beta testing comprised a slightly larger group (N=19, mean age 37.37 years, SD 9.73; range 22-68 years, 68.4% female) and focused on eliciting user feedback (via email) post testing. During both the test studies, individuals were asked to keep a hardcopy record of any data submissions so that app data could be checked for accuracy. Average time to submit drinking information during testing was 34 seconds.

Suggestions provided by individuals involved in this phase and that were incorporated into the final version of the app included making an instructional video explaining how to use CNLab-A available to participants [44], providing post experimental summary feedback regarding total standard drinks consumed and average daily intake, along with several minor changes to the app user interface. Otherwise, testers indicated that compliance with app protocols was not onerous. They all reported using a mix of real-time and prompt-based submissions.

The requirements analysis and feature and interface design phases took almost 4 months, whereas the alpha and beta testing of the app implementation phase spanned approximately 8 weeks. We had 3 meetings with the programmer in the early stages of development and then a further meeting toward the completion of the feature and interface design phase. All other communication was via email. The final production version of the app was eventually made available on both iOS (8.4+) and Android (Kitkat 4.1+) platforms. As operating systems evolve, both apps will be audited to ensure they continue to function as required.

### Participants and Procedure

**Figure 2:**
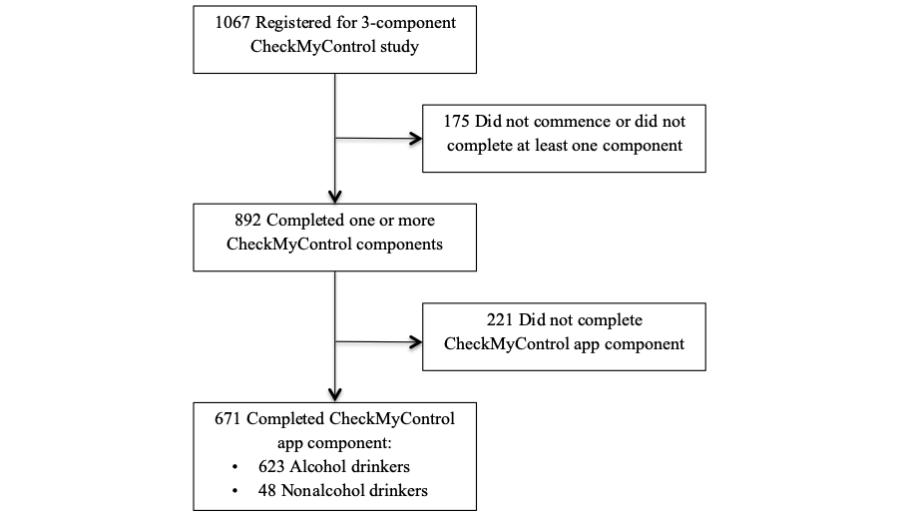
Flow diagram following Consolidated Standards of Reporting Trials guidelines of study participation.

This study was based on data from 671 participants (mean age 23.12 years, SD 7.24; range 16-56 years, 70% female) that form a subset of an ongoing project—entitled CheckMyControl—investigating the relationship between alcohol use and various social and cognitive factors in the healthy population. This subsample completed the app component of the project (Figure 2). Participants were recruited via adverts posted in and around the University of Melbourne, researcher networks, and social media posts; as such, they formed a convenience sample. Individuals were eligible to take part in this study if they were fluent in English and aged 13 years or older. The University of Melbourne Human Research Ethics Committee approved the study in accordance with the standards for ethical research of the National Health and Medical Research Council.

After reading a plain language statement and providing informed consent, participants answered a Web-based researcher-devised demographic survey and the Alcohol Use Disorders Identification Test (AUDIT) [39]. The demographic survey included questions pertaining to country of birth, first language, and place of residence. Participants were also asked if they had ever been diagnosed with an alcohol or substance use disorder. Participants were then required to download and use the CNLab-A smartphone app to record alcohol use for 21 days.

They were compensated Aus $10 for time spent completing Web-based surveys and Aus $0.50 each day information about alcohol consumption was submitted via the app (regardless of whether alcohol had been consumed or not). Participants received a bonus Aus $9.50 if app data were submitted on all 21 days. The maximum participants could be reimbursed was Aus $30.

### Measures

The AUDIT is a 10-item screening measure that asks participants to respond to questions assessing alcohol intake, problems, and dependence with reference to the preceding 6 months. Participants were categorized into hazard (n=286) and nonhazard (n=385) groups based on their score, with scores of 8 or more indicative of hazardous alcohol consumption [45]. Previous studies have shown the AUDIT is a valid instrument for assessing alcohol misuse among adolescents—sensitivity (Sn)=.71; specificity (Sp)=.84—university students (Sn=.75-.89; Sp=.73-.82), and the general population (Sn=.92; Sp=.94) in various countries, including Australia (Sn=.93; Sp=.82) [39,46-48].

CNLab-A is a freely available custom-built app that can be used to record alcohol intake for research purposes. Once downloaded, CNLab-A requires participants to allow it to send them notifications. One notification is preset to 8 am, whereas the other can be set to suit the user. Although participants are directed at the outset to record alcohol consumption as it happens (or as soon thereafter as possible), notifications serve to prompt individuals to input information twice daily in case they neglect to do so when drinking. Thus, alcohol intake data can be submitted at any time, either in response to notifications or while drinking. A unique identification code, provided to participants during the Web-based component of the study, is required before the app opens. CNLab-A has previously been found to be a valid measure of alcohol intake [27].

### Statistical Analyses

Independent *t* tests and Chi-square analyses were conducted to determine whether hazard and nonhazard groups were matched demographically. Homogeneity of variance was assessed using Levene Test, where this assumption was violated, adjusted *t* values and associated degrees of freedom were reported. Effect sizes were computed for *t* tests using *r* values; they were interpreted according to Cohen guidelines: 0.10=small, 0.30=moderate, and 0.50=large effect [49].

Participants were considered compliant with app protocols if they responded to at least 1 notification each day for the 21-day experimental period. We assessed both number of days of use and submissions per day as a function of gender, age bracket (13-19 years, 20-29 years, and ≥30 years), and hazard and nonhazard group status using *t* tests and 1-way analysis of variance. Reactivity was assessed using linear growth model analyses, conducted using SPSS 24 (IBM). These analyses examined (1) change in drinks consumed each day and (2) change in daily (“yes”) responding over the 21-day experimental period as a function of AUDIT group membership. In the first analysis, drinks per day (within persons; level 1) were nested within individuals (between persons; level 2); in the second, daily (“yes”) response rate (within persons; level 1) was nested within individuals (between persons; level 2). Time was centered on the first day of data collection (Day 0); each unit of time represented an interval of 1 day [50]. All mixed models were estimated using restricted maximum likelihood [51]. Initially, slope variance and autocorrelation were included in both models, but they were removed when parameter estimates for these effects were found to be very small (<0.01).

## Results

### Descriptive Statistics

At the time of testing, 39.9% (268/671) of the sample was aged under 20 years, 46.2% (310/671) was aged 20 to 29 years, and 13.9% (93/671) was aged 30 years or over. In Australia, individuals in the 18 to 29-year-old age bracket are more likely than others to consume alcohol in a manner that increases their short-term risk of alcohol-related injury, whereas almost 19% are at risk of long-term alcohol-related harm [52]. Most participants were born in Australia (66.8%, 448/671), spoke English as their first language (80.0%, 537/671), and lived in urban regions (88.1%, 591/671). Census data show 67% of the Australian population is locally born and 79% speak English as their first language [53]. Most Australians (86%) reside in urban regions [54]. A small number of participants indicated they identified as an Indigenous Australian (0.6%, 4/671). This was less than what the census data suggest as typical for the state (Victoria) in which this study took place (0.8%) [53]. However, this difference is likely a product of our recruitment campaign. As advertisements for the study were posted in and around the University of Melbourne, the sample contained a large number of young tertiary-aged participants (85.7%, 575/671). The proportion of indigenous students studying at this institute in 2016 stood at 0.6% [55].

Before commencing the 21-day recording period, the minority of participants (2.2%, 15/671) indicated they had never consumed alcohol; the majority (92.8%, 623/671) described themselves as regular drinkers. A small proportion indicated they had been diagnosed with an alcohol (0.3%, 2/671) or substance (0.4%; 3/671) use disorder. Hazard and nonhazard groups did not differ significantly with regard to age, *t*_669_=1.02, *P*=.31 or years of education, *t*_654.11_=1.65, *P*=.10. There was a significant association between gender and AUDIT group membership, χ^2^_671_=5.96, *P*=.02. The odds of being a hazardous drinker were 1.51 times higher for males than females (95% CI 0.22-2.01). There were significant differences between the AUDIT scores of the hazard (mean 12.45, SD 4.30) and nonhazard (mean 4.64, SD 3.59) groups, *t*_547.67_=24.94, *P*<.001. Mean alcohol intake indices recorded via CNLab-A are detailed in Table 2.

### Compliance

On average, participants used CNLab-A 20.27 (SD 1.88) days out of 21 (96.5%); 96.0% (644/671) of participants completed at least 1 submission per day for the entire 21-day experimental period. There were no significant differences as a function of gender *t*_669_=0.83, *P*=.41, or age bracket, *F*_2,668_=0.34, *P*=.71, with regard to number of days of use. Moreover, there were no differences between hazard and nonhazard groups, *t*_669_=0.69, *P*=.49. As data submission was either event- or notification-contingent, there was no upper limit to the number of drinking sessions participants could report using the app. Participants received a maximum of 42 notifications asking them to record information about their drinking. They submitted data, on average, 2.00 (SD 0.41) times per day.

With regard to the average number of responses per day, there were no significant differences as a function of gender, *t*_669_=0.65, *P*=.51, or age bracket, *F*_2,668_=1.08, *P*=.34. In addition, there were no differences on the basis of AUDIT group membership, *t*_669_=0.61, *P*=.54. There were 27,355 data points captured via the app in total.

**Table 2:** Average alcohol intake indices as recorded via CNLab-A app (21 days), including hazard (n=286) and nonhazard (n=385) group totals and differences.

Drinking indices	Total, mean (SD)	Hazard, mean (SD)	Nonhazard, mean (SD)	*t*_669_^a^	95% CI	Pearson correlation *r*
Days drinking	5.32 (4.17)	6.90 (4.02)	4.15 (3.90)	8.91	2.14-3.35	.33
Total drinks^b^	24.26 (25.41)	37.45 (28.09)	14.47 (17.75)	12.16	19.27-26.70	.43
Drinks per day	1.20 (1.25)	1.86 (1.38)	0.71 (0.86)	12.45	0.97-1.33	.43
Drinks per day drinking	3.98 (3.02)	5.53 (2.92)	2.83 (2.56)	12.45	2.27-3.12	.43
Hourly rate of drinking	2.20 (2.09)	2.60 (2.14)	1.89 (2.00)	4.41	0.40-1.04	.17
Highest drink count in 2 hours	4.14 (3.15)	6.03 (3.37)	2.79 (2.09)	14.34	2.80-3.69	.48
4/4+ intake^c^	2.16 (2.58)	3.48 (2.83)	1.19 (1.86)	11.89	1.91-2.67	.42
6/6+ intake	1.31 (1.92)	2.26 (2.27)	0.61 (1.20)	11.14	1.35-1.94	.40
8/8+ intake	0.85 (1.44)	1.51 (1.70)	0.36 (0.94)	10.41	0.94-1.38	.37
12/12+ intake	0.31 (0.80)	0.58 (1.06)	0.10 (0.43)	7.26	0.35-0.61	.27
20/20+ intake	0.07 (0.31)	0.12 (0.41)	0.03 (0.20)	3.55	0.04-0.15	.14

^a^*P* values all <.001.

^b^Drinks refer to self-reported alcohol consumption in Australian standard drinks (1 drink=10 g alcohol).

^c^4/4+ (and so forth) intake refers to occasions where 4 or more drinks were consumed in 1 episode.

**Table 3:** Parameter estimates for linear growth model of drinks per day as a function of hazard and nonhazard Alcohol Use Disorders Identification Test group membership.

Parameter		Estimate (SE)	*t/z* ^a^	*df*	*P* value	95% CI
**Fixed effects (intercept, slopes)**						
	Intercept (Day 0)	0.67 (0.08)	8.68	2301.73	<.001	0.52 to 0.82
	Time	–0.01 (0.01)	–1.80	13418.00	.07	–0.02 to 0.001
	Hazard	1.48 (0.11)	13.26	2301.73	<.001	1.26 to 1.70
	Hazard by time	–0.03 (0.01)	–3.50	13418.00	<.001	–0.04 to–0.01
**Random effects ([co-]variances)**						
	Between-person (level 2) intercept	0.74 (0.06)	12.28	—^b^	<.001	0.63 to 0.87
	Within-person (level 1) residual	7.54 (0.09)	81.91	—	<.001	7.37 to 7.73

^a^*t* test value for fixed effects parameters; Wald z value for random effects parameters.

^b^Not applicable.

**Table 4:** Parameter estimates for linear growth model of daily (“yes”) responses per day as a function of hazard and nonhazard AUDIT group membership.

Parameter		Estimate (SE)	*t/z* ^a^	*df*	*P* value	95% CI
**Fixed effects (intercept, slopes)**						
	Intercept (Day 0)	0.32 (0.02)	18.24	982.85	<.001	0.28 to 0.35
	Time	–0.003 (0.001)	–3.63	13418.00	<.001	–0.004 to –0.001
	Hazard	0.15 (0.03)	5.99	982.85	<.001	0.10 to 0.20
	Hazard by time	–0.004 (0.001)	–3.53	13418.00	<.001	–0.006 to –0.002
**Random effects ([co-]variances)**						
	Between-person (level 2) intercept	0.08 (0.01)	16.86	—^b^	<.001	0.07 to 0.09
	Within-person (level 1) residual	0.14 (0.002)	81.91	—	<.001	0.14 to 0.15

^a^*t* test value for fixed effects parameters; Wald z value for random effects parameters.

^b^Not applicable.

### Reactivity

Table 3 details parameter estimates for fixed and random effects of the linear growth analysis model for change in drinks consumed each day. With regard to fixed effects, the nonhazard group reported consuming significantly fewer standard drinks (0.67) on Day 0 than the hazard (0.67+1.48=2.15) group. The nonhazard group showed no significant decrease in consumption over the 21-day experimental period, whereas the hazard group demonstrated a slight but significant decrease in drinking over the same period (0.04 standard drinks per day). Within-person variance (7.54) equates to 2.75 SD units; thus, 95% of observed residuals were between ±5.49 units of their fitted values. The intercept variance (0.74) corresponds to 0.86 SD units; therefore, 95% of the population varied between ±1.72 units of the typical intercept for their group (Table 3).

Table 4 shows parameter estimates for fixed and random effects of the linear growth model for change in daily (“yes”) responses. The nonhazard group responded (“yes”) significantly less often (0.32) on Day 0 than the hazard (0.32+0.15=0.47) group. A slight but significant decrease in responding was evident for both the nonhazard (–0.003 per day) and hazard (–0.003+(– 0.004)=–0.007 per day) groups. Within-person variance (0.14) equates to 0.37 SD units; thus, 95% of observed residuals were between ±0.75 units of their fitted values. The intercept variance (0.08) corresponds to 0.28 SD units; therefore, 95% of the population varied between ±0.57 units of the typical intercept for their group (Table 4).

## Discussion

### Principal Findings

In this study, we aimed to describe the development and implementation of a custom-built smartphone app devised to measure real-time (or near real-time) alcohol consumption behavior in the healthy population. Designed for use in the research arena, the app was a product of an iterative process that included elements typical of agile software design. Decisions made during each phase of development were informed by a desire to create an app that the participants could download onto their own smartphones without ever having to visit the lab. We anticipated this might minimize disadvantages—such as equipment and training costs, participant burden, and missing data—often associated with using some types of electronic protocols, while simultaneously enhancing benefits pertaining to sample size and diversity. We additionally explored methodological factors related to app protocol compliance and reactivity as a function of hazard and nonhazard drinker status.

Compliance with app protocols was high. Participants were required to submit data about their drinking (regardless of whether they had consumed alcohol or not) at least once per day for 21 days. On average, they uploaded data on 96.5% of days, and there were no differences between hazard and nonhazard groups with regard to the number of days of app use or the number of responses per day. Presumably, the use of daily payments and an end-of-study bonus (for 21 consecutive days of data submissions) incentivized responding. In previous alcohol intake-based studies utilizing various electronic methods of collecting data—including SMS text messaging [56], hand-held electronic devices [57], and interactive voice response systems [8]— incentivized responding resulted in similarly high rates of compliance. It should be noted that when the measure is freely available via marketplace vendors, the size of the sample can quickly balloon. As such, it is important to not only balance the burden of protocol compliance with the incentive offered but it is also necessary to consider overall budget constraints and ensure there are some swift means of limiting access to the app if required.

Our results suggest there was some slight degree of reactivity—particularly among hazardous drinkers—to the app protocol. Though the effect was small, the hazard group decreased their intake significantly over the experimental period: 0.80 standard drinks in 21 days, which represented a 2% decrease in total standard drinks for this group. By contrast, nonhazard drinkers showed no significant reduction in consumption over the same period. This accords with evidence from other studies demonstrating some reduction in alcohol consumption only because of measurement among hazardous drinkers [28,29]. Even though participants received no feedback about their drinking during the assessment period, it is possible that those in the hazard group were motivated to modify their intake because the act of recording it made them more aware of their behavior. Equally, the knowledge that they were being monitored may have induced them to drink less. Considered a manifestation of the Hawthorne effect, social desirability is thought to underpin this type of assessment reactivity [58]. It is also possible that reductions in consumption were the result of satisficing; that is, participants may have responded “yes” to drinking less often over time to avoid having to submit further information via the app. Both hazard and nonhazard groups showed a significant reduction in the frequency of responding “yes” to drinking over the 21-day period. However, the rate of this reduction was very small: nonhazard participants decreased “yes” responding by 0.06 and hazard participants by 0.14 in 21 days. As such, this reduction might be a reflection of increased familiarity with the app over time, rather than satisficing; that is, participants may have summarized their drinking across a day into fewer submissions once they became more familiar with the app.

There is some debate in the literature regarding reactivity to real-time measures. Several investigators postulate such assessment reduces the likelihood of reactivity related to social desirability as participants record data in the absence of the researcher [31]. Bates and Cox found participants were, for example, more likely to reveal lifetime alcohol consumption details when they completed surveys outside, as opposed to inside, the lab [59]. Other researchers speculate real-time methods are particularly susceptible to reactivity effects because assessments are completed in close proximity to the behavior, giving participants time to consider their actions [1], though it has also been suggested that repeated surveying may reduce reactivity via habituation [60,61]. Nonetheless, there is consensus that reactivity is generally an overlooked facet of real-time research and further investigation is required [21,31,62]. It is possible, for instance, that reactivity differs according to the population. Our finding that hazardous drinkers reacted to the app protocol to a greater extent than nonhazard drinkers would certainly support this supposition. Our data also suggest app-based alcohol-related intervention studies would benefit from the inclusion of a measurement-only control condition to disentangle the effects related to reactivity from those linked to the intervention.

### Limitations

Several limitations to this study must be noted. Although representative of the population at large in terms of country of birth, first language, and usual place of residence, young female university students predominated in our sample. Although current research suggests men and women are equally likely to download and use health-orientated smartphone apps [63,64], women in Australia typically drink less than men and are consequently at decreased risk of alcohol-related harm [52]. Nonetheless, our hazard group reported drinking alcohol a third of the time during the experimental period and, on average, consumed in excess of 5 standard drinks per episode. According to Australian guidelines, this pattern of consumption places them at increased risk of both short-term alcohol-related injury and lifetime harm from alcohol [65]. Notwithstanding, future studies would be required to determine if similar rates of app compliance and reactivity are evident in a sample dominated by young men. Similarly, older individuals might respond in different ways to app-based protocols designed to assess alcohol consumption behavior. A recent survey found 59% of Australians over 65 years are willing to use or already utilize technology designed to track health [66]. In addition to research showing age has not been identified as a barrier to participation in mental health studies [67], this suggests older individuals might respond well to alcohol-related research apps. Moreover, as age-related illness and associated difficulties pertaining to condition severity, transportation, and inconvenience do inhibit participation in research [67], apps people access via marketplace vendors and download to their own smartphones may possibly boost participation in this age group. Future studies would nonetheless be required to fully consider app compliance and reactivity among older individuals. Finally, we did not examine if different assessment periods impact compliance and reactivity in diverse ways. A shorter experimental period might, for instance, diminish the effect of reactivity, though this may limit how effectively the app captures variability of intake. This is another potential area for further research.

### Conclusions

In conclusion, we examined the feasibility of developing and employing an app—downloaded by participants to their own smartphones—designed to collect alcohol intake information for research purposes. We demonstrate how utilizing apps such as CNLab-A can yield a potentially large sample representative of the population. Both hazard and nonhazard participants appeared highly compliant when using app protocols. Although there was some evidence of reactivity in our study, especially among hazardous drinkers, effect sizes were small. Our findings suggest the CNLab-A app—or potentially 1 similar—is a methodologically sound means of examining alcohol consumption behavior across time. In future, such apps can be paired with those that chart cognition, affect, or social and environmental factors in real time to facilitate a more thorough investigation of the antecedents and consequences of drinking behavior.

## Abbreviations

AUDIT: Alcohol Use Disorders Identification Test
BAC: blood alcohol content
SMS: short message service
Sn: sensitivity
Sp: specificity
